# Supplementary material for: Pelvic floor muscle training associated with the photobiomodulation therapy for women affected by the genitourinary syndrome of menopause: a study protocol
Source: PeerJ. 2024 Nov 29;12:e17848. doi: 10.7717/peerj.17848 (PMC11610460; doi:10.7717/peerj.17848)
Supplement: Supplemental Information 1 [file peerj-12-17848-s001.docx]

**Test instruments**

**Pelvic floor manometry**

<https://www.ncbi.nlm.nih.gov/pmc/articles/PMC5662229/pdf/pone.0187045.pdf>

**International Consultation on Incontinence Questionaire – short Form (ICIQ – SF)**

<https://www.scielo.br/j/rsp/a/sJjtsdfRRnmcgBSLB6gGqDx/?lang=en>

**Female Sexual Function Index**

<https://www.scielo.br/j/rbgo/a/bF7SYs4SbxJV4FjZZFSC3vP/abstract/?lang=pt>

**Utian Quality of Life**

<https://www.scielo.br/j/rbgo/a/spkLRcKjSY8WvBTjjRhVS5g/>

**Global impression of improvement and Modified Oxford scale**

Attached to the e-mail

**Vaginal Health Index**

<https://www.aafp.org/pubs/afp/issues/2000/0515/p3090.html>
