# Supplementary material for: Pelvic floor muscle training associated with the photobiomodulation therapy for women affected by the genitourinary syndrome of menopause: a study protocol
Source: PeerJ. 2024 Nov 29;12:e17848. doi: 10.7717/peerj.17848 (PMC11610460; doi:10.7717/peerj.17848)
Supplement: Supplemental Information 2 [file peerj-12-17848-s002.pdf]

Validation of two global impression questionnaires for incontinence

Ilker Yalcin, PhD, and Richard C. Bump, MD
Indianapolis, Ind

OBJECTIVE: The purpose of this study was to assess the construct validity of two global assessment questions, the Patient Global Impression of Severity and of Improvement, in female patients with stress urinary incontinence.
STUDY DESIGN: This was a secondary analysis of data from two double-blind, placebo-controlled studies that evaluated duloxetine for the treatment of predominant stress urinary incontinence in the United States (n = 1133 patients). Assessment variables included incontinence episode frequency, the Incontinence Quality of Life Questionnaire results, fixed volume (400 mL) stress pad test results, and the Patient Global Impression of Improvement and of Severity question results.
RESULTS: Spearman correlation coefficients were 0.36, 0.20, and –0.50 among the Patient Global Impression of Severity question and incontinence episode frequency, stress pad test, and Incontinence Quality of Life Questionnaire results, respectively (all $P < .0001$). Mean incontinence episode frequency and median stress pad test results increased and mean Incontinence Quality of Life Questionnaire results decreased with increasing Patient Global Impression of Severity question severity levels. Similarly, significant ($P < .0001$) correlations were observed between the Patient Global Impression of Improvement question response categories and the three independent measures of improvement in stress urinary incontinence (0.49, 0.33, and –0.43 with incontinence episode frequency, stress pad test, and Incontinence Quality of Life Questionnaire results, respectively). As with the Patient Global Impression of Severity question, differences in mean changes for Incontinence Quality of Life Questionnaire and median percent changes for incontinence episode frequency and stress pad test among the Patient Global Impression of Improvement question response categories were highly significant ($P < .0001$). These relationships indicate appropriate and significant associations between the Patient Global Impression of Severity and of Improvement questions and the three independent measures of stress urinary incontinence severity and improvement, respectively.
CONCLUSION: The Patient Global Impression of Severity and of Improvement question responses were correlated significantly with incontinence episode frequency, stress pad test, and Incontinence Quality of Life Questionnaire measures, which established the construct validity of these two global assessment questions for baseline severity and treatment response, respectively. (Am J Obstet Gynecol 2003;189:98-101.)

Key words: Stress urinary incontinence, Global Assessment Questionnaire, quality of life, duloxetine

Quality of life has been promoted increasingly as an outcome domain that should be assessed in incontinence treatment trials, especially for treatments such as medications or behavioral interventions that are more likely to improve than totally cure incontinence. Several committees that have been charged with the establishment of standards for outcomes research that is related to urinary incontinence and other pelvic floor disorders have specifically recommended that the domain of "quality of life" be assessed with condition-specific instruments.[1-5] The second International Consultation on Incontinence has evaluated, rated, and recommended multiple incontinence-specific quality-of-life instruments that assess symptom severity, distress, and impact.[6] Although these instruments are accepted as critical components of incontinence research, most of the instruments are relatively long and often require both time and calculations to derive a score that is not interpretable instinctively for clinical treatment. In contrast, global indexes that ask an individual patient to rate the severity of a specific condition (single-state scales) or to rate the response of her condition to therapy (transition scales) are simple, direct, easy to use, and intuitively understandable to the clinician.[7]

The scientific and clinical goal of a global scale is to get an overall appraisal of a complex phenomenon, not

From Lilly Research Laboratories.
Supported by Eli Lilly and Company.
Presented at the Twenty-Third Annual Meeting of the American UroGynecologic Society, San Francisco, Calif, October 6-118, 2002.
Reprint requests: Ilker Yalcin, Eli Lilly and Company Corporate Center, DC 6114, Indianapolis, IN 46285. E-mail: yalcin@lilly.com
© 2003, Mosby, Inc. All rights reserved.
0002-9378/2003 $30.00 + 0
doi:10.1067/mob.2003.379

Table I. Patient Global Impression of Improvement (PGI-I) Scale

Check the one number that best describes how your urinary tract condition is now, compared with how it was before you began taking medication in this study.	
1.	Very much better
2.	Much better
3.	A little better
4.	No change
5.	A little worse
6.	Much worse
7.	Very much worse

Table II. Patient Global Impression of Severity (PGI-S) Scale

Check the one number that best describes how your urinary tract condition is now.	
1.	Normal
2.	Mild
3.	Moderate
4.	Severe

to evaluate every component part of the phenomenon. Although a multiple-question condition-specific quality-of-life instrument that evaluates many components of a phenomenon can be used to generate a total score that purports to be an overall appraisal of the condition, this appraisal is limited by the fact that it is ultimately derived from questions that were developed by "experts" rather than from the unique personal perception of the individual patient.[8] Global indexes avoid this limitation, but at the cost of uncertainty regarding the precise aspects of the manifestations of the disease severity or improvement that resulted in an individual patient selecting a specific rating.[7] Thus, there is an imprecision in a global scale because it has no intrinsic operational criteria for demarcating the ratings; this results in a degree of imprecision across the spectrum of different people who might use it. Nonetheless, global ratings can be quite precise when applied by the same person over time.[7] Gill and Feinstein[8] recommend the use of a single global index because it "can reflect the disparate values and preferences of individual patients (and) offer investigators the most overtly sensible approach to measure quality of life."

Although many multiple-question condition-specific quality-of-life instruments have been developed and validated adequately for incontinence research,[6] no condition-specific global instrument has been developed or evaluated. The aim of this study was to establish the construct validity of two single-question global indexes for urinary incontinence by correlating them with several other measures of incontinence severity: incontinence episode frequency recorded on real-time diaries, total score on the Incontinence Quality of Life questionnaire (I-QOL), and grams of fluid lost in a standardized stress pad test. This validation of the Patient Global Impression of Severity (PGI-S; a single-state scale) and the Patient Global Impression of Improvement (PGI-I, a transition scale) scales represents a secondary analysis of data from two randomized controlled trials that examined the safety and efficacy of duloxetine (a balanced serotonin-norepinephrine reuptake inhibitor) in women with stress urinary incontinence (SUI).

Methods

Participants. Women with urinary incontinence of at least 3 months duration were enrolled in two double-blind, placebo-controlled, randomized clinical trials. The first trial was a phase II dose finding study (duloxetine 20, 40, and 80 mg/d) that was conducted at 48 study centers in the United States, the details of which have been previously published (n = 553 patients).[9] The second study was a phase III study that was conducted in United States (n = 580 patients, 52 study centers) and Canada (n = 103 patients, 7 study centers) to evaluate the efficacy of duloxetine 80 mg/d.[10] The case definition for both studies included a predominant symptom of SUI, the lack of predominant symptoms of enuresis or urge urinary incontinence; diurnal and nocturnal frequencies of <8 and <3, respectively; on screening history, negative funnel infusion cystometry with a first sensation of >100 mL and a bladder capacity >400 mL; and a positive fixed volume cough stress test and stress pad test. Women with greater than stage II pelvic organ prolapse were excluded from the study. The dose-finding study required at least four episodes per week to be reported by the subject; the second study required at least seven episodes on a screening diary, which resulted in a more severe group of subjects.

The institutional review board for each site approved the study, and written informed consent was obtained from all participants.

Study design. In both studies, after a 2-week no-drug lead-in period followed by a 2-week blinded placebo lead-in period, subjects were assigned randomly under double-blind conditions to 12 weeks of acute treatment with duloxetine or placebo. Subjects were seen at 4-week intervals throughout the acute treatment period.

Baseline and outcome measurements. The primary severity measure at baseline and during treatment was the incontinence episode frequency, recorded real-time on daily diaries for 1 week before each baseline and treatment visit. Another efficacy measure at baseline and during treatment was the I-QOL, a 22-item validated condition-specific questionnaire.[11,12] The I-QOL includes questions that evaluate both the distress and impact of urinary incontinence, with a score of 100 being the best possible and 0 being the worst possible quality of life. It is one of the two quality-of-life instruments that received the Second International Consultation on Incontinence highest ("highly recommended") rating for

Table III. Summary of incontinence episode frequency, stress pad test, and I-QOL at each PGI-S response category at baseline

PGI-S responses	*No. (%)*	*Mean incontinence episode frequency (SD)*	*Median stress pad test (g) (minimum, maximum)*	*Mean I-QOL (SD)*
Normal	96 (8.5)	12.3 (9.1)	15 (2, 399)	68.1 (20.4)
Mild	410 (36.5)	14.3 (12.0)	9 (2, 313)	72.6 (13.5)
Moderate	530 (47.2)	21.7 (16.1)	19 (2, 414)	57.9 (16.3)
Severe	88 (7.8)	32.8 (20.3)	29 (2, 397)	36.6 (15.6)
P value between group		<.0001	<.0001	<.0001
Spearman ρ (*P* value)		.36 (<.0001)	.20 (<.0001)	–.50 (<.0001)

Table IV. Summary of incontinence episode frequency, stress pad test, and I-QOL at each PGI-I response category

PGI-I Responses	*No. (%)*	*Change in incontinence episode frequency (%)*	*Change in stress pad test (%)**	*Change in I-QOL (SD)*
Very much better	134 (12.3)	–92	–86	+19.1 (14.8)
Much better	230 (21.1)	–63	–51	+13.2 (13.9)
A little better	277 (25.4)	–46	–27	+6.3 (10.2)
No change	389 (35.6)	–19	–1	+3.8 (9.7)
A little worse	47 (4.3)	+12	+23	–0.1 (9.0)
Much worse	12 (1.1)	+54	+4	–5.9 (15.6)
Very much worse	2 (0.2)	+500	Not available	–13.1 (28.1)
P values between group		<.0001	<.0001	<.0001
Spearman ρ (*P* value)		.49 (<.0001)	.33 (<.0001)	–0.43 (<.0001)

*Postbaseline stress pad test result was obtained only in the dose finding study.

condition-specific measures of incontinence impact in men and women.[6]

The PGI-I (Table I) was administered at each of the three postrandomization visits. Two measures were also determined during the baseline prerandomization period, the PGI-S (Table II) and the fixed bladder volume (400 mL) stress pad test.[9] Only the dose-finding study included the stress pad test at the last postrandomization visit.

Statistical analysis. Both studies were sized to have a power of at least 80% to detect a treatment difference between duloxetine and placebo of 20% in the median percent change in weekly incontinence episode frequency with an overall type I error of 0.05.

The construct validity of PGI-S was assessed by an examination of the relationship among the four PGI-S response categories and the three baseline measurements (incontinence episode frequency, I-QOL, and stress pad test). Statistical tests were performed to evaluate the differences among PGI-S categories for each of the three assessments. Between-group differences were tested using one-way analysis of variance (when means are presented) and its nonparametric alternative Kruskal-Wallis (when medians are presented). In addition, the Spearman correlation coefficient was used to evaluate the degree of association between PGI-S and the other three measures.

The construct validity of the PGI-I was assessed by a correlation of the last postrandomization PGI-I response to the median percent changes from baseline to postrandomization for incontinence episode frequency and stress pad test and the mean absolute changes from baseline to postrandomization in I-QOL, all at the final study visit. Among-group differences in PGI-I categories were also tested using the methods described for the PGI-S analysis. Medians rather than means were used to summarize baseline stress pad test and percent changes from baseline to postbaseline in stress pad test and incontinence episode frequency because of the extreme outliers. Percent changes in incontinence episode frequency and stress pad test values were used to standardize changes in these variables across their considerable range of baseline severity. Because the range of I-QOL severity at baseline was much narrower, absolute changes in this variable were analyzed. All statistical analyses were done using the Statistical Analysis System, version 8.0, software (SAS Institute, Cary, NC).

Results

A total of 1133 women entered the trials in the United States and were assigned randomly to receive duloxetine 20 mg/d (n = 138 women), duloxetine 40 mg/d (n = 137 women), duloxetine 80 mg/d (n = 433 women), or placebo (n = 425 women) therapy. Overall, the mean age was 51.1 ± 9.8 (SD) years, and the mean body mass index was 29.3 ± 6.5 kg/m^2. Most of the subjects (91%) were white. None of these characteristics differed significantly

among treatment groups. Self-reported ratings of urinary tract function using the PGI-S were 8.5% normal, 36.5% mildly abnormal, 47.2% moderately abnormal, and 7.8% severely abnormal. Means for incontinence episode frequency, I-QOL, and stress pad test at the baseline visit were 19.1 per week and 62.4 and 41 g, respectively. The median stress pad test was 16 g, which indicated several high-end outliers (range, 2-414 g).

Table III shows baseline incontinence episode frequency, stress pad test, and I-QOL data for each PGI-S response category. The mean incontinence episode frequency and median fluid loss on stress pad test were greater and mean I-QOL scores were lower with increasing PGI-S severity category. Comparisons among PGI-S categories showed significant differences for all three variables. All Spearman correlation coefficients between PGI-S and the other three variables were statistically significant ($P < .0001$).

Table IV lists the outcome data by PGI-I response category and shows significant ($P < .0001$) correlations between the PGI-I response categories and all three of the other measures of improvement in SUI. As with the PGI-S, PGI-I category differences for all three other improvement variables were highly significant ($P < .0001$).

Comment

The data from these combined studies indicate that the PGI-S and PGI-I responses were correlated significantly with incontinence episode frequency, stress pad test, and I-QOL measures, which established the construct validity of the 2 global assessment indexes for baseline severity and for treatment response, respectively, in a population of women in the United States with SUI. The PGI-I and PGI-S were modeled after scales that were described previously and used successfully in psychopharmacologic research.[13] The stems were altered for use in patients with lower urinary tract conditions, although the response options are identical to the older instruments.

It is important to emphasize that the construct validity that was demonstrated in this analysis is applicable only to a population of women with predominant SUI. The applicability of the PGI-S and PGI-I for men or women with other lower urinary tract symptoms or conditions has not been established.

It is interesting that the relationships between worsening measures of SUI and increased perceived functional abnormalities with the use of the PGI-S are not profound between the normal/mildly abnormal response categories. This implies that, at the mild end of the SUI spectrum, individual perceptions are more important than incontinence severity in the determination of a woman's appraisal of her condition. A similar, although less profound, phenomenon is observed in the no-change/a little worse categories of the PGI-I.

We conclude that these global indexes are a valuable addition to stress incontinence research and are capable of reflecting a woman's overall appraisal of her condition and her response to treatment, while taking into account the disparate values and preferences of individual patients.[8] At the same time, these two global indexes help us make an assessment as to how other commonly used quantitative measures, whose values do not translate directly into an understanding of self-perceived incontinence severity, relate to patients' global impressions. For instance, we can estimate how many weekly stress incontinent episodes must occur before a woman classifies her incontinence as moderate or severe or how a baseline I-QOL score of 50 translates into the patient's perception of severity. In a similar way, we can say that a patient begins to perceive herself as being better with treatment when her incontinence episodes decrease by about 45% or her I-QOL score improves by 6 or 7 points.

REFERENCES

1. Donovan J, Naughton M, Gotoh M, Corcos J, Jackson S, Kelleher C, et al. Symptom and quality of life assessment. In: Abrams P, Shoury S, Wein A, editors. Incontinence. Plymouth, UK: Health Publication; 1999. p. 295-332.
2. Mattiasson A, Peters T, Schaefer W, Blaivas J, Jonas U, Stanton S, et al. Research methodology in incontinence. In: Abrams P, Shoury S, Wein A, editors. Incontinence. Plymouth, UK: Health Publication; 1999. p. 893-929.
3. Mattiasson A, Djurhuus JC, Fonda D, Lose G, Nordling J, Stohrer M. Standarization of outcome studies in patients with lower urinary tract dysfunction: a report on general principles from the Standardisation Committee of the International Continence Society. Neururol Urodyn 1998;17:249-53.
4. Lose G, Fantl JA, Victor A, Walter S, Wells TL, Wyman J, et al. Outcome measures for research in adult women with symptoms of lower urinary tract dysfunction. Neurourol Urodyn 1998;17:255-62.
5. Wall LL, Norton P, Versi E, Bump R. Evaluating the outcome of surgery for pelvic organ prolapse. Am J Obstet Gynecol 1998;178:877-9.
6. Donovan JL, Badia X, Corcos J, Gotoh M, Kelleher C, Naughton M, et al. Symptom and quality of life assessment. In: Abrams P, Cardozo L, Khoury S, Wein A, editors. Incontinence. 2nd ed. Plymouth, UK: Health Publication; 2002. p. 267-316.
7. Feinstein AR. Global indexes and scales. In: Clinimetrics. New Haven (CT): Yale University Press; 1987. p. 91-103.
8. Gill TM, Feinstein AR. A critical appraisal of the quality of quality-of-life measurements. JAMA 1994;272:619-26.
9. Norton PA, Zinner NR, Yalcin I, Bump RC. Duloxetine versus placebo in the treatment of stress urinary incontinence. Am J Obstet Gynecol 2002;187:40-8.
10. Zinner NR, Dmochowski RR, Norton PA, Miklos JR, Yalcin I, Bump RC. Duloxetine versus placebo in the treatment of stress urinary incontinence (SUI). Neurourol Urodyn 2002;21:383-4.
11. Wagner TH, Patrick DL, Bavendam TG, Martin ML, Buesching DP. Quality of life of persons with urinary incontinence: development of a new measure. Urology 1996;47:67-71.
12. Patrick DL, Martin ML, Bushnell DM, Yalcin I, Wagner TH, Buesching DP. Quality of life of women with urinary incontinence: further development of the incontinence quality of life instrument (I-QOL). Urology 1999;53:71-6.
13. Guy W. ECDEU assessment manual for psychopharmacology. Rockville (MD): National Institute of Mental Health, US Department of Health, Education, and Welfare; 1976. p. 218-22.
