# Supplementary material for: Pelvic floor muscle training associated with the photobiomodulation therapy for women affected by the genitourinary syndrome of menopause: a study protocol
Source: PeerJ. 2024 Nov 29;12:e17848. doi: 10.7717/peerj.17848 (PMC11610460; doi:10.7717/peerj.17848)
Supplement: Supplemental Information 3 [file peerj-12-17848-s003.pdf]

ELSEVIER

Physiotherapy

Physiotherapy 99 (2013) 207–211

Oxford Grading Scale vs manometer for assessment of pelvic floor strength in nulliparous sports students

T. Da Roza [a], T. Mascarenhas [c], M. Araujo [b], V. Trindade [a], R. Natal Jorge [a,*]

[a] *IDMEC, Faculty of Engineering, University of Porto, Porto, Portugal*
[b] *Department of Sport Gynaecology, Escola Paulista de Medicina, Universidade Federal de São Paulo, São Paulo, Brazil*
[c] *Department of Gynaecology and Obstetrics, Hospital de S. João, Faculty of Medicine of Porto University, Porto, Portugal*

Abstract

Objectives To compare pelvic floor muscle strength in nulliparous sports students measured using the modified Oxford Grading Scale and a Peritron manometer; and to compare the manometric measurements between continent and incontinent subjects.
Design Cross-sectional study. All subjects were evaluated twice on the same day; first by vaginal digital examination and subsequently by vaginal pressure using a Peritron manometer.
Participants Forty-three nulliparous female sports students [mean age 21 (standard deviation 4) years] from the Sports Faculty of the University of Porto.
Results This study found a significant moderate correlation between the Oxford Grading Scale score and peak pressure on manometry ($r = 0.646$, $P = 0.002$). Mean maximal strength for the entire group was 70.4 cmH_2O (range 21 to 115 cmH_2O). Out of 43 subjects, 37% ($n = 16$) demonstrated signs of incontinence. On manometry, no significant differences were found in vaginal resting pressure or peak pressure between the continent and incontinent groups.
Conclusions There was moderate correlation between peak pressure on manometry and the Oxford Grading Scale score. Peritron manometer measurements of pelvic floor muscle contractions showed no significant differences in vaginal resting pressure and peak pressure in continent and incontinent subjects.
© 2012 Chartered Society of Physiotherapy. Published by Elsevier Ltd. All rights reserved.

Keywords: Oxford Grading Scale; Pelvic floor muscles; Sports students; Manometer; Nulliparous; Urinary incontinence

Introduction

Pelvic floor muscles (PFM) play an important role in the maintenance of continence and support of the pelvic organs [1]. Correct contraction of the PFM causes elevation and occlusion of the urogenital hiatus to resist downward forces during increased intra-abdominal pressure. Lack of contraction or delayed or weak contraction of the PFM may lead to urinary incontinence [2]. Urinary incontinence is defined by the International Continence Society (ICS) as the complaint of any involuntary loss of urine [3].

A correct PFM contraction is characterised by an anterior and cephalad movement [1]. Several studies have shown that PFM exercise is effective to treat urinary incontinence in women [4,5]. Thus, it is essential for physiotherapists conducting PFM rehabilitation programmes to measure function and strength [6].

Due to the location of the PFM inside the pelvis, it is difficult to observe PFM function. Hence, vaginal palpation is often used to evaluate muscle strength, and also to teach patients how to perform a correct contraction [7]. Several vaginal palpation rating scales have been used in clinical practice [6], but the most common in physiotherapy seems to be the modified Oxford Grading Scale [8,9].

Maximum strength is measured through a maximum voluntary contraction (MVC), where an individual attempts to recruit as many muscle fibres as possible to develop force [10]. Instruments such as manometers can be used to provide objective data for the evaluation of PFM strength.

* Correspondence: Rua Dr Roberto Frias, s/n; 4200-465 Porto, Portugal. Tel.: +351 225081491; fax: +351 22 557 41 70.
E-mail address: rnatal@fe.up.pt (R.N. Jorge).

0031-9406/$ – see front matter © 2012 Chartered Society of Physiotherapy. Published by Elsevier Ltd. All rights reserved.
http://dx.doi.org/10.1016/j.physio.2012.05.014

Table 1
Baseline characteristics in both groups.

Variable	Continent group ($n=27$)	Incontinent group ($n=16$)	P-value
Age (years)	19.9 (1.7)	22.8 (5.9)	0.131[b]
Body mass index (kg/m^2)	21.1 (1.7)	21.1 (2.2)	0.997[a]
Age at menarche (years)	12.4 (1.1)	12.6 (1.3)	0.494[a]

Data are presented as mean (standard deviation).
[a] Independent sample *t*-test.
[b] Mann–Whitney test.

The primary aim of this study was to compare PFM strength in nulliparous sports students measured using the modified Oxford Grading Scale and a Peritron manometer. The secondary aim was to compare the manometric measurements between continent and incontinent subjects.

Methods

Participants

This study received ethical approval from São João Hospital Ethical Committee, Porto, Portugal. Forty-three nulliparous female sports students [mean age 21 (standard deviation 4.0) years] from the Sports Faculty of the University of Porto participated in the study, and provided written consent. The sample characteristics are summarised in Table 1. All the subjects performed high levels of physical activity, classified according to the International Physical Activity Questionnaire – Short Form [11].

The subjects were recruited via questionnaires, briefed (verbal and written explanations) about the nature of the study, and screened for eligibility. Exclusion criteria included being pregnant, previous pelvic surgery, neurological problems, ongoing urinary tract infections, diagnosis of pelvic organ prolapse, or inability to contract the PFM properly.

Procedure

Each subject answered a two-part questionnaire. The first part was designed to investigate demographic characteristics, and the second part was the International Consultation on Incontinence Questionnaire – Short Form (ICIQ–SF) [12]. The ICIQ–SF is a clinical instrument used to assess the presence or absence of urinary incontinence, developed by the ICS and translated and validated for the Portuguese language [13].

Clinical evaluation

The subjects were taught to contract their PFM as strongly as possible and then to relax completely. PFM contraction was assessed by digital examination of the vagina using the five-point Oxford Grading Scale: 0, no contraction; 1, flicker; 2, weak; 3, moderate; 4, good; and 5, strong. This was performed using two fingers, with the two distal phalanges inside the introitus vagina. After digital examination, vaginal pressure was measured at rest and at MVC using a Peritron manometer (Cardio Design, Victoria, Australia). For both methods of assessment, three consecutive squeezes were recorded with a 10-second interval between efforts [7]. Two measurements were taken on the same day with a 1-hour interval.

All measurements were performed in a crook lying position, and subjects were asked to contract their PFM as hard as possible; the best of three contractions was registered. Co-contraction of the gluteal, hip adductor or rectus abdominal muscles was discouraged through previous instruction. To ensure valid measurement during the examination, no visible contraction of the other muscles was allowed. Only contractions with simultaneous observable inward movement of the perineum were considered valid [14].

Statistical analyses

To analyse the differences in baseline characteristics between the groups, the independent sample *t*-test was used to compare parametric data, and Mann–Whitney's *U*-test was applied to data that demonstrated a non-normal distribution. Spearman's correlation test was used to compare the values obtained with the Peritron manometer and the modified Oxford Grading Scale. $P<0.05$ was considered to indicate statistical significance.

Results

Fifty-five female sports students were initially recruited into the study and signed an informed consent form. However, one subject was pregnant, six had never had sexual intercourse, and five could not perform a proper PFM contraction. Thus, the final sample consisted of 43 nulliparous sports students.

For the entire study sample, digital assessment of PFM strength using the Oxford Grading Scale system was distributed as follows: weak ($n=1$), moderate ($n=6$), good ($n=10$) and strong ($n=26$). Manometer measurements revealed an average score of 70.4 cmH_2O (range 21 to 115 cmH_2O). There was a significant moderate positive correlation between the Oxford Grading Scale score and peak pressure on manometry ($r=0.646$, $P=0.002$). Fig. 1 shows the correlation between the Peritron manometer measurements and the Oxford Grading Scale scores.

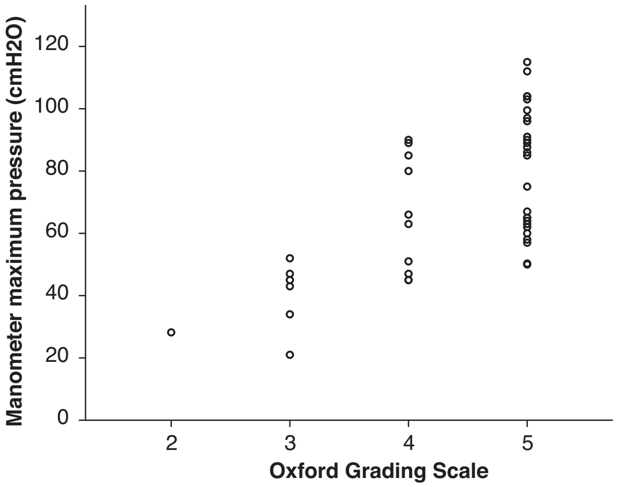

Fig. 1. Peritron manometer measurements vs Oxford Grading Scale scores.

Table 2
Type of urinary incontinence among nulliparous sports students (International Consultation on Incontinence Questionnaire–Short Form), $n = 16$. Multiple answers possible.

	n
Loss of urine during coughing/sneezing	4/16
Loss of urine during physical activity	9/16
Loss of urine before reaching the toilet	3/16
Loss of urine after finishing urination and once dressed	3/16
Loss of urine for no obvious reason	0
Loss of urine when asleep	0
Loss of urine all the time	0

Out of 43 subjects, 37% ($n = 16$) displayed signs of incontinence, as revealed by the first question on the ICIQ–SF. Of these 16 subjects, five reported urge incontinence, nine reported stress incontinence and two reported mixed urinary incontinence (Table 2).

Table 3 shows mean vaginal pressure at rest and during MVC (cmH_2O) for both groups. No significant differences were found between the two groups. The MVC manometer readings suggested that the incontinent group had weaker PFM than the continent group; however, this difference was not statistically significant.

Discussion

This study found a high prevalence of urinary incontinence symptoms in nulliparous female sports students. This is in agreement with previous survey studies conducted by Bo *et al.* [15]. Previous studies using manometer evaluation demonstrated that intravaginal balloon devices could produce reliable pressure recordings when measuring PFM function through vaginal pressure measurement [14]. The results from studies evaluating inter-rater reliability of other palpation scoring systems and squeeze pressure using Spearman's correlation rho ranged from $r = 0.60$ to $r = 0.90$ [16–18]. Isherwood and Rane performed a blinded comparison of PFM strength assessed by perineometer and Oxford Grading Scale digital examination, and found good agreement [kappa = 0.73, 95% confidence interval (CI) from 0.67 to 0.79] in a sample of 263 women [19].

Correlation analysis is used to determine whether the values of two variables are associated. Spearman's correlation was used to determinate the degree of linear correlation between the two sets of data. This is appropriate when different methods are used to measure one construct, and the question is how two measurements obtained by different instruments co-vary [20].

However, Bland and Altman illustrated that a strong correlation does not necessarily indicate agreement between the two sets of data [21]. The present study used the values reported by Richman *et al.* to indicate the strength of the relationship between variables (0.80 to 1.00, high reliability; 0.60 to 0.80, moderate reliability; <0.59, questionable reliability). As such, moderate correlation was found between digital evaluation of PFM strength using the Oxford Grading Scale and readings of maximum squeeze pressure obtained with a manometer (Spearman's rho 0.65) (Fig. 1).

The results of this study suggest acceptable reliability, although Fig. 1 illustrates that for every grade defined by the Oxford Grading Scale, there is a relatively wide distribution of pressures measured by the manometer, indicating that these scores are not interchangeable. However, other studies only showed fair inter-rater reliability for the Oxford Grading Scale using Cohen's kappa (0.37), despite a higher Spearman's rho value [7,22]. This may be due to differences in the calculation of Spearman's rho and Cohen's kappa where the former is an indication of linear association without respect to scaling (data can demonstrate perfect correlation where one set of data is double the values of the other set of data) where kappa is a measure of agreement.

Frawley *et al.* tested intratester reliability of the Peritron manometer, and found intraclass correlation coefficients for squeeze pressure readings of 0.95, 0.91, 0.96 and 0.92 for crook lying, supine, sitting and standing positions, respectively. They reported high reliability of MVC measured by the Peritron manometer [23]. Additionally, a recent study also showed moderate inter-rater reliability [22].

A concern in all studies of this nature is whether the device in question is isolating and measuring the desired parameter. It seems that this issue is of particular importance for manometers as the PFM form one wall of the abdominopelvic cavity.

As highlighted by Hundley *et al.*, it is not claimed that manometers are able to isolate pelvic floor musculature as precisely as other more technically elaborate devices, but a degree of imprecision does not preclude their value as measurement devices [17]. As the increase in abdominal pressure will affect urethral, vaginal and rectal pressures, creating a downward movement, some authors have suggested that a valid measurement can be ensured by simultaneous observation of inward movement of the perineum [24,25]. In the

Table 3
Mean vaginal resting pressure and during maximal voluntary contraction (cmH_2O) in continent and incontinent groups.

Measurement	Continent group ($n = 27$)	Incontinent group ($n = 16$)	P-value
Vaginal resting pressure	37.7 (11)	36.3 (14)	0.465[a]
Maximal voluntary contraction	72.6 (24.3)	66.6 (23.2)	0.497[a]

Data are presented as mean (standard deviation).
[a] Mann–Whitney test.

current study, correct PFM contraction was obtained through both proper instruction and observation.

Vaginal digital examination is a low-cost method and is relatively easy to conduct, representing advantages over the use of a manometer. Vaginal digital examination can be recommended as a good technique for physiotherapists to understand, teach and give feedback to patients about the correctness of PFM contraction. Clear correlation between weak PFM strength during MVC and urinary incontinence symptoms has not been established.

The bladder neck receives support from a strong and toned PFM, resistant to stretching, thereby limiting its downward movement during effort and exertion, preventing leakage of urine. This PFM tone can be reported by vaginal resting pressure measurements. Bø *et al.*, in an uncontrolled magnetic resonance imaging reconstruction study, showed a significant reduction in the internal surface area of the levator ani after PFM training, suggesting an increase in passive stiffness of the levator ani, which is indicative of the state of PFM tone [24]. In contrast with this study, dynamometric studies have shown that women with urinary incontinence demonstrate less PFM tone and maximal strength compared with continent women [26]. The present study found no differences in vaginal resting pressure and PFM strength between the continent and incontinent groups. The small sample size and the large standard deviation may explain why no significant difference in PFM strength was found between the groups.

Factors contributing to urinary incontinence in young nulliparous women are not fully understood. In this study, most subjects were incontinent during physical activity ($n = 9$). A heavy training routine could be a risk factor for urinary incontinence due to the increase in intra-abdominal pressure and the impact from ground reaction forces, which could contribute to stress urinary incontinence.

A strength of this study was the use of a Peritron manometer, which has been shown to have very good reliability and moderate inter-rater reliability in a homogeneous group of young, nulliparous sports students. Limitations of this study include the small sample size; type II error may have occurred, particularly when considering the difference in the MVC measurements between continent and incontinent subjects. Moreover, the assessors for vaginal digital examination and manometer readings were not blinded, and the study had a non-randomised design. Another limitation of this study is that the findings may only be valid for young nulliparous sports students, and the results may differ in older women with pelvic floor disorders.

Conclusion

This study found moderate correlation between digital evaluation of PFM contraction strength and Peritron manometer measurements. No differences in vaginal resting pressure or MVC pressure were found between the continent and incontinent groups. Further studies with young, nulliparous sports students are needed to support these findings.

Ethical approval: Ref. No. #2010/09/30. São João Hospital Ethical Committee, Porto, Portugal.

Conflict of interest: None declared.

References

[1] Retzky SS, Rogers Jr RM. Urinary incontinence in women. Clin Symp 1995;47:2–32.
[2] Bump RC, Norton PA. Epidemiology and natural history of pelvic floor dysfunction. Obstet Gynecol Clin N Am 1998;25:723–46.
[3] Haylen BT, de Ridder D, Freeman RM, Swift SE, Berghmans B, Lee J, *et al.* An International Urogynecological Association (IUGA)/International Continence Society (ICS) joint report on the terminology for female pelvic floor dysfunction. Int Urogynecol J Pelvic Floor Dysfunct 2010;21:5–26.
[4] Morkved S, Salvesen KA, Bø K, Eik-Nes S. Pelvic floor muscle strength and thickness in continent and incontinent nulliparous pregnant women. Int Urogynecol J Pelvic Floor Dysfunct 2004;15:384–9, discussion 90.
[5] Bø K. Pelvic floor muscle strength and response to pelvic floor muscle training for stress urinary incontinence. Neurourol Urodyn 2003;22:654–8.
[6] Bø K, Sherburn M. Evaluation of female pelvic-floor muscle function and strength. Phys Ther 2005;85:269–82.
[7] Bø K, Finckenhagen HB. Vaginal palpation of pelvic floor muscle strength: inter-test reproducibility and comparison between palpation and vaginal squeeze pressure. Acta Obstet Gynecol Scand 2001;80:883–7.
[8] Messelink B, Benson T, Berghmans B, Bø K, Corcos J, Fowler C, *et al.* Standardization of terminology of pelvic floor muscle function and dysfunction: report from the pelvic floor clinical assessment group of the International Continence Society. Neurourol Urodyn 2005;24: 374–80.
[9] Laycock J. Clinical evaluation of pelvic floor. In: Schussler B, Laycock J, Norton P, Stanton S, editors. Pelvic floor re-education: principles and practice. London: Springer-Verlag; 1994. p. 42–8.
[10] Knuttgen HG, Kraemer WJ. Terminology and measurement in exercise performance. J Appl Sports Sci Res 1987;1:1–10.
[11] Craig CL, Marshall AL, Sjostrom M, Bauman AE, Booth ML, Ainsworth BE, *et al.* International physical activity questionnaire: 12-country reliability and validity. Med Sci Sports Exerc 2003;35:1381–95.
[12] Avery K, Donovan J, Peters TJ, Shaw C, Gotoh M, Abrams P. ICIQ: a brief and robust measure for evaluating the symptoms and impact of urinary incontinence. Neurourol Urodyn 2004;23:322–30.

[13] Tamanini JT, Dambros M, D'Ancona CA, Palma PC, Rodrigues Netto Jr N. [Validation of the "International Consultation on Incontinence Questionnaire – Short Form" (ICIQ-SF) for Portuguese]. Rev Saude Publ 2004;38:438–44.
[14] Bø K, Kvarstein B, Hagen R, Larsen S. Pelvic floor muscle exercise for the treatment of female stress urinary incontinence: II. Validity of vaginal pressure measurements of pelvic floor muscle strength and the necessity of supplementary methods for control of correct contraction. Neurourol Urodyn 1990;9:479–87.
[15] Bø K, Stien R, Kulseng-Hanssen S, Kristofferson M. Clinical and urodynamic assessment of nulliparous young women with and without stress incontinence symptoms: a case–control study. Obstet Gynecol 1994;84:1028–32.
[16] Hove MCPS, Pool-Goudzwaard AL, Eijkemans MJC, Steegers-Theunissen RPM, Burger CW, Vierhout ME. Face validity and reliability of the first digital assessment scheme of pelvic floor muscle function conform the new standardized terminology of the International Continence Society. Neurourol Urodyn 2009;28:295–300.
[17] Hundley AF, Wu JM, Visco AG. A comparison of perineometer to brink score for assessment of pelvic floor muscle strength. Am J Obstet Gynecol 2005;192:1583–91.
[18] Brink CA, Wells TJ, Sampselle CM, Taillie ER, Mayer R. A digital test for pelvic muscle strength in women with urinary incontinence. Nurs Res 1994;43:352–6.
[19] Isherwood PJ, Rane A. Comparative assessment of pelvic floor strength using a perineometer and digital examination. BJOG 2000;107:1007–11.
[20] Armitage P, Berry G, Matthews JNS. Statistical methods in medical research. 4th ed. Oxford: Blackwell Publishing; 2002.
[21] Bland JM, Altman DG. Statistical methods for assessing agreement between two methods of clinical measurement. Lancet 1986;1:307–10.
[22] Ferreira CH, Barbosa PB, de Oliveira Souza F, Antonio FI, Franco MM, Bø K. Inter-rater reliability study of the modified Oxford Grading Scale and the Peritron manometer. Physiotherapy 2011;97:132–8.
[23] Frawley HC, Galea MP, Phillips BA, Sherburn M, Bø K. Reliability of pelvic floor muscle strength assessment using different test positions and tools. Neurourol Urodyn 2006;25:236–42.
[24] Bø K, Berghmans B, Morkved S, Van Kampen M. Evidence based physical therapy for the pelvic floor: bridging science and clinical practice. 1st ed. Oxford: Elsevier; 2007.
[25] Bump RC, Mattiasson A, Bø K, Brubaker LP, DeLancey JO, Klarskov P, *et al.* The standardization of terminology of female pelvic organ prolapse and pelvic floor dysfunction. Am J Obstet Gynecol 1996;175:10–7.
[26] Morin M, Bourbonnais D, Gravel D, Dumoulin C, Lemieux MC. Pelvic floor muscle function in continent and stress urinary incontinent women using dynamometric measurements. Neurourol Urodyn 2004;23:668–74.

Available online at www.sciencedirect.com
SciVerse ScienceDirect
